# The effect of information on prostate cancer screening decision process: a discrete choice experiment

**DOI:** 10.1186/s12913-020-05327-x

**Published:** 2020-05-26

**Authors:** M. Charvin, G. Launoy, C. Berchi

**Affiliations:** 1 Normandie Univ, UniCaen, Inserm, Anticipe, 14000 Caen, France; 2 University Hospital of Caen, Caen, France

**Keywords:** Prostate cancer screening, Health education material, Discrete choice

## Abstract

**Background:**

Prostate cancer screening is controversial because of uncertainty about its benefits and risks. The aim of this survey was to reveal preferences of men concerning prostate cancer screening and to test the effect of an informative video on these preferences.

**Methods:**

A stated preferences questionnaire was sent by e-mail to men aged 50–75 with no history of prostate cancer. Half of them were randomly assigned to view an informative video. A discrete choice model was established to reveal men’s preferences for six prostate cancer screening characteristics: mortality by prostate cancer, number of false positive and false negative results, number of overdiagnosis, out-of-pocket costs and recommended frequency.

**Results:**

A population-based sample composed by 1024 men filled in the entire questionnaire. Each attribute gave the expected sign except for overdiagnosis. The video seemed to increase the intention to abstain from prostate cancer screening.

**Conclusions:**

The participants attached greater importance to a decrease in the number of false negatives and a reduction in prostate cancer mortality than to other risks such as the number of false positives and overdiagnosis. Further research is needed to help men make an informed choice regarding screening.

## Background

Cancer screening participation is dependent on how people assess the benefit-risk ratio. Individual characteristics like cognitive skills, emotions and a priori beliefs with regard to screening affect this assessment [1]. At the population level, the main benefit of screening is frequently evaluated by randomized trials that take the reduction in global and specific mortality as an endpoint. To date, prostate cancer screening has been highly controversial within the medical community because of the absence of certitude that prostate cancer mortality is reduced by screening. Moreover, the reduction is relatively small (1 in 1.000 men screened regularly) [2–4]. Regarding this limited benefit, the negative effects of screening procedure are especially due to the slow evolution of prostate cancer. In many cases, this results in overdiagnosis and overtreatment but also to other risks due to the technical limitations of PSA screening, i.e. risk of false positive and false negative results. False positive results induce unnecessary biopsies.

Prostate cancer screening consists of assaying prostate specific antigen (PSA) in the blood and a digital rectal examination. About one third of the French male population aged from 50 to 69 years old received at least one PSA assay during the year 2014 [5]. In France, the test is prescribed in most cases by general practitioners (GP). They may refer the patient to a urologist in the event of an abnormal test result. Subsequently, the urologist may perform a prostate biopsy according to the patient’s history and preferences. Biopsies are necessary to diagnose prostate cancer (PC).

Prostate cancer screening is not recommended by French health authorities [6] but is promoted by a urologist association. There is no national prostate cancer-screening program organized by health authorities in France, given the benefit-risk ratio [6]. Nevertheless, screening may be performed at the patient’s request after a discussion with his GP. PSA dosage and GP consultation are partially covered by national health insurance (respectively 60% of 10.80 € and 16.50 € on 25.00€). National and international (e.g. United States Preventive Services Task Force [7]) recommendations encourage informed choice and shared decision-making for prostate cancer screening so that men may choose to receive screening or not according to their individual preferences.

To achieve this goal, GPs must provide enough information on prostate cancer screening and make sure that men understand its pros and cons (e.g. false positives, overdiagnosis), medical statistics and uncertainty [8, 9]. To help healthcare providers to meet this objective [10–12], a growing number of decision aids and educational tools on prostate cancer screening are being developed. These tools can have an effect on men’s intention to be screened and on their understanding of the issues involved. In France, some institutions make printed brochures available for use by clinicians (e.g. French National Cancer Institute (2016), ARC foundation (2014)). A research project in 2015 evaluated the effect on participation of a two-page decision aid [13]. They found a reduction in stated screening participation in the intervention arm. In their decision aid, they did not use key words and omitted some risks (e.g. false negative results). Screening efficacy was shown by means of an icon array to facilitate understanding of the risks. However, the data presented on screening efficacy from the European Randomized Study of Screening for Prostate Cancer (ERSPC) are not the most recent. Moreover, in some studies on education tools, videos have demonstrated their superior communicative potential over other modes of communication such as internet website pamphlets and routine consultations [14]. Thus, we developed a new video on prostate cancer screening in order to help men make an informed choice.

We tested its effect on the process of choosing to undergo prostate cancer screening or not. We also investigated quantitatively men’s preferences regarding the benefits and risks of screening.

## Method

To investigate men preferences, we performed a discrete choice experiment (DCE), an econometric method increasingly used in health economics [15].

### Discrete choice experiment

DCEs allow fictive screening program characteristics to be ranked according to their relative importance in the decision. The method is based on Lancaster’s consumer theory [16], which stipulates that a program or an intervention in healthcare can be described by its main characteristics, called attributes, and their relative levels. In a DCE, the respondent states which alternative he/she prefers among the fictive scenarios. These scenarios are composed of several attributes (e.g. efficacy of the test, out-of-pocket costs, etc.) and differ according to several levels of attribute. Preferences are extracted from the respondent’s stated choices.

### Identification and selection of attributes and levels

To implement a stated DCE, attributes and levels are selected and fictive scenarios are created. Attributes and levels were chosen to test the effect of the benefit-risk ratio on prostate cancer screening choices. We performed PubMed and Econlit searches in May 2018 to identify attributes and corresponding levels using keywords: “discrete choice” and “cancer screening”. Four DCE were found to explore preferences with regard to prostate cancer screening with discrete choice analysis [17–20]. Attributes used in these studies were related to death from prostate cancer [17–20], recommended screening frequency [17], number of biopsies [19] and PSA false positive results [17, 18], number or percentage of prostate cancers diagnosed [18–20], risk of overdiagnosis [20], risk of overtreatment [17], treatment side-effects (impotence and incontinence) [18–20] and out-of-pocket costs [17, 18, 20]. False negative results were not introduced into these choice models. We interviewed 5 experts (i.e. epidemiologist, ethicist, health economist and physicians) to select and formulate the attributes and levels. They based their choice on key objective elements (e.g. available care strategies, benefits and risks of each procedure), which should be provided by GPs during a consultation. Finally, six attributes were selected: risk of mortality by prostate cancer, risk of false positive results, risk of false negative results, risk of overdiagnosis, recommended screening test frequency and out-of-pocket costs.

Out-of-pocket costs for the patient only concern medical expenses related to a cancer screening procedure (i.e. GP consultation, and PSA blood assay). In France, since 2017, a routine consultation GP is charged € 23,00 of which € 16,50 are reimbursed by the health insurance system and up to € 5,50 by the patient’s private insurance policy. A PSA assay costs € 10,80 of which 60% is reimbursed by the health insurance system and up to € 3,32 by private insurance. Levels of out-of-pocket cost attribute vary according to these rates and to the various fictive reimbursement rates applied by the social security system, i.e. from no reimbursement at all to complete reimbursement of medical expenses.

Since there is no national prostate cancer screening program in France, the frequency of the PSA assay and the rectal examination depends on the GP. Levels associated with the recommended frequency attribute were based on the frequency tested during the main surveys and on GPs’ prescription habits.

Levels of the four risk attributes were extracted from the major clinical trials on prostate cancer screening (ERSPC [3], PLCO and CAP [4]) [3, 21–25]. Based on recent progress in risk communication [26], the wording of attribute levels based on risks was established with the same indicator (i.e. per 1000 persons regularly screened). Table 1 gives an overview of the attributes and levels used in this study. Respondents could obtain more details about the attribute definitions (i.e. false positive rate, false negative rate, overdiagnosis, out-of-pocket costs) by clicking on the attribute’s label.

**Table 1:** Attributes and levels of fictitious prostate cancer screening programs

Attributes	Attribute label used in survey	Attribute supplementary information	Levels	Opt-out option level	References
**Mortality by prostate cancer**	Number of deaths by prostate cancer		2 / 1000^a^ 5 / 1000^a^ 6 / 1000^a^	6 / 1000	Schröder et al.(2014) [3]
**False positive result**	Number of false positive results to the screening test (false alarm)	This wrong alert induces potentially useless supplementary exams (biopsies) because men do not have cancer	50 / 1000^a^ 150 / 1000^a^ 250/ 1000^a^	0	Kipeläinen, et al. (2011) [25]
**False negative result**	Number of false negative results on screening test	Prostate cancer is undetected yet individual has prostate cancer	1 / 1000 5 / 1000 10 / 1000	0	Verbeek, et al. (2018) [24]
**Overdiagnosis**	Number of prostate cancers detected, even treated unnecessarily (overdiagnosis)	This prostate cancer would never cause symptoms, pain or death	10 / 1000^a^ 30 / 1000^a^ 50 / 1000^a^	0	Etzioni, et al. (2013) [22]
**Recommended frequency**	Frequency at which you should be screened		Every year Every 2 years Every 4 years	NA	Tsodikov et al. (2017) [2]
**Out-of-pocket costs**	Amount to pay for each screening session	Amount is not reimbursed by national health insurance or supplementary health insurance	0 € 10 € 20 € 40 €	0	NABM [1], NGAP [2]

Notes: ^a^per one thousand men regularly screened for prostate cancer

^(1)^http://www.codage.ext.cnamts.fr/codif/nabm/index_presentation.php?p_site=AMELI

^(2)^https://www.ameli.fr/sites/default/files/Documents/377680/document/ngap_14.04.18.pdf

Table 1.

**Fig. 1:**
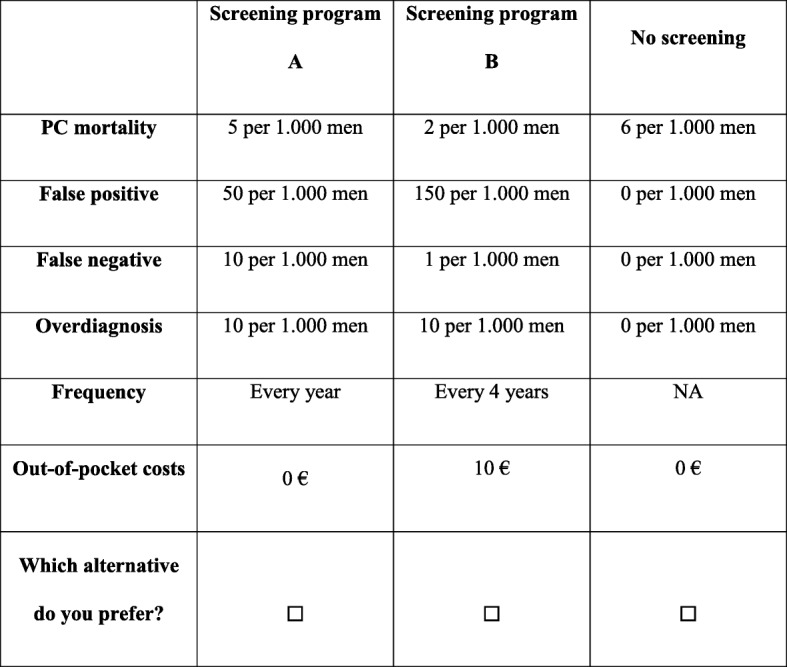
Example of a choice set

Given the selection of attributes and levels, 972 combinations (4^1^*3^5^) were available for this survey. To reduce the cognitive effort caused by too many tasks per respondent, an experimental design was created to obtain 14 scenarios by using the OPTEX procedure in the SAS software (version 9.4) [27]. In a second time of the procedure, scenarios were paired to obtain 7 choice situations with 2 screening alternatives. This fractional nearly orthogonal design maximized the D-efficient score (90.0788). Alternative scenarios extracted from this design were randomly distributed between two fictive screening options by applying a blocking strategy [28]. At the end, a total of 7 tasks per respondent was obtained. Figure 1

### Study design and questionnaire

Before completing the questionnaire developed for this study (available in the supplementary files), half of the respondents had access to a 6-min video on prostate cancer screening produced by our research team. A simple randomization was performed to determine this access. Several patients, a urologist and GPs watched a previous version of the video and suggested changes to improve its clarity and neutrality and to limit its cognitive demand. The video is available in the supplementary files. The video started with information on prostate anatomy and physiology. Then, key epidemiological data on prostate cancer were illustrated with diagrams. Next, the screening procedure was presented. Its benefit and risks were graphically represented with two icon arrays (consequences for 1.000 men with and without screening) as it is used by The Canadian Task Force on Preventive Health Care [29], for example. This format is recommended for communicating about risks and benefits, especially among men with a low level of numeracy [30]. At the end, the official French guidelines were explained to the participants.

Either directly or after the video, each participant received instructions on the stated preference experiment (i.e. context of prostate cancer screening, background to DCE). Respondents had to express their intentions regarding hypothetical and fictive screening programs. In this experiment, the participants chose one of two prostate cancer screening programs. Since it is irrelevant to force respondents to choose between screening programs without proposing an option without screening if that is their preference [31], choice sets included two fictive prostate cancer screening programs and one opt-out option (i.e. “do not undergo a prostate cancer screening test”). An opt-out option is an alternative whose attribute levels do not change according to the choice situations. Figure 1 is an example of a choice situation proposed to respondents. A within-set dominated-pairs test was added to test the rationality of DCE responses [32, 33]. In this choice situation, a dominated screening alternative was less interesting for each attribute level. This dominated alternative is composed by a screening program more frequent, more expensive, with more risks and less efficacy. Respondent characteristics likely to influence choice of cancer screening participation were also collected (e.g. age, prostate cancer screening experience, highest level of education). The last question concerned difficulty in completing the questionnaire (from easy to hard).

A pilot study in 50 respondents tested the relevance of the attributes, the level of comprehension and the feasibility of the full questionnaire. A few changes were made to the introductory section after this phase. The mean survey duration was 17 min, including viewing.

### Study sample

**Fig. 2:**
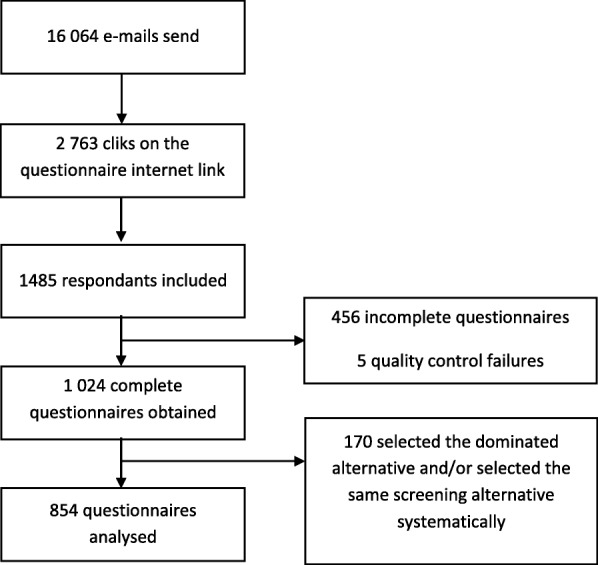
Flow chart

A survey institute oversaw the recruitment process. They performed a sampling approach with the quota method to be representative of the male population aged from 50 to 75 years old and without any prostate cancer diagnosis. For this purpose, criteria used were age, French regions, type of urban agglomeration, and socio-professional categories. In January 2019, the survey institute used e-mail (16,064 emails sent) to contact potential respondents from a French panel. Among the recipients, 2.703 men clicked on the study link. If respondents agreed with the terms, they could complete the online survey. Finally, a total of 1.024 respondents completed the entire questionnaire. Figure 2

Two tests were included to evaluate choice rationality. The dominant alternative of the within-set dominated pairs test was chosen by 62.21% of respondents. One hundre seventy men failed the rationality test and/or systematically selected the same screening alternative, whatever the screening scenario content. They were excluded from the analysis. Finally, statistical analysis was performed on data from 854 participants. Among them, 427 respondents had to watch the video before completing the questionnaire.

In accordance with French law, ethical committee (CLERS: Comité Local d’Ethique de la Recherche) and CNIL (Commission Nationale de l’Informatique et des Libertés) approval was obtained before the survey began.

### Statistical analyses

Based on the maximization of utility principle, the relative importance of the choice components could be estimated through alternative utility functions. In these utility functions, utility is explained by a measurable part composed of attributes. All attributes were included in a logistic model in the SAS software (version 9.4) as continuous variables. The main effect model for an individual n and a choice alternative j is presented below:

$$ {\mathrm{U}}_{\mathrm{nj}}={\upbeta}_0+{\mathrm{ASC}}_{\mathrm{opt}-\mathrm{out}}+{\upbeta}_1\mathrm{x}\ {\mathrm{DR}}_{\mathrm{nj}}+{\upbeta}_2\mathrm{x}\ {\mathrm{FP}}_{\mathrm{nj}}+{\upbeta}_3\mathrm{x}\ {\mathrm{FN}}_{\mathrm{nj}}+{\upbeta}_4\mathrm{x}\ {\mathrm{OD}}_{\mathrm{nj}}+{\upbeta}_5\mathrm{x}\ {\mathrm{CO}}_{\mathrm{nj}}+{\upbeta}_6\mathrm{x}\ {\mathrm{FR}}_{\mathrm{nj}}+{\upvarepsilon}_{\mathrm{nj}}. $$

Where β_0_ is the Alternative Specific Constant (ASC) representing choice parameters unmeasured, ASC _opt-out_ is another alternative specific constant which is equal to 1 if the no-screening option is chosen, 0 otherwise. DR _n j_, FP _nj_, FN _nj_, OD _nj_, CO _nj_, FR _nj_ are vectors of the attributes mortality by prostate cancer, false positive result rate, false negative result rate, overdiagnosis rate, out-of-pocket costs and recommended frequency of screening, β_1_, β_2_, β_3,_ β_4_, β_5_, and β_6_ their vector of parameters, and Ɛ_nj_ represents the random and unobservable part. We assumed that the latter component was independently and identically distributed (i.i.d.).

A ranking of attribute importance in men’s choices is then available with the sign and the magnitude of each coefficient. A priori expectations had a negative impact on alternative utility for all attributes.

### Willingness to pay

Marginal Willingness-To-Pay (MWTP) was then calculated from out-of-pocket costs and risk attributes. For example, MWTP represents how much men were willing-to-pay in order for an additional man not to succumb to prostate cancer per 1000 men screened $\frac{\upbeta\ \mathrm{DR}}{-\upbeta\ \mathrm{CO}}$. Confidence intervals of these estimations were estimated by using the delta method [34], which stipulated that the confidence interval of WTP

$$ \mathrm{WTP}\pm {\mathrm{z}}_{\alpha /2}\ \sqrt{\mathit{\operatorname{var}}(WTP)}. $$

Interactions with individual characteristics, anxiety and video.

Various specifications of the model were tested by incorporation different interaction components like socio-demographic data. Health anxiety level was broken down into three levels (i.e. low, medium and high) according to terciles. A high level of anxiety was hypothesizedto reinforce the negative estimation of mortality by prostate cancer, false negative, false positive and overdiagnosis attributes. Men with a high level of anxiety were also hypothesized to increase the value of screening.

Video access was also added as an interaction term to test our hypothesis that an informative video could modify choice preferences. The video was hypothesized to improve understanding of the benefits and risks of screening and thus to reinforce the negative effect of mortality by prostate cancer, false positive rate, false negative rate and overdiagnosis. It was also hypothesized to reduce the positive perception of screening by representing the benefit-risk ratio of prostate cancer screening or the statement by the French health authorities.

## Results

### Description of study population

Characteristics of the study sample and associated statistics are presented in Table 2. Mean age of the sample was 61.33 ys (s.d. 6.91). Self-estimated health was considered as good or very good by 45.32% of the population. About 15% of the population declared *feeling afraid that they may have cancer* often or most of the time and 18.03% knew someone with prostate cancer. About 90% of the population had a regular follow-up with a GP and less than 10% with an urologist.

**Table 2:** Characteristics summary of men in sample

	Video access (***n*** = 427)	No video access (***n*** = 427)	Difference (Chi-2)	Total (***n*** = 854)
**Age** (mean = 61.34, s.d. = 6.945)				
50–62	245 (57.38)	230 (53.86)		475 (55.62)
62–75	182 (42.62)	197 (46.14)	0.3015	379 (44.38)
**Education level**				
Low (≤ high school diploma)	236 (55.27)	217 (50.82)		453 (53.04)
High (> high school diploma)	191 (44.73)	210 (49.18)	0.1927	401 (46.96)
**GP follow-up (more than 1 per year)**				
Yes	382 (89.46)	379 (88.76)		761 (89.11)
No	45 (10.54)	48 (11.24)	0.7417	93 (10.89)
**Urologist follow-up (more than 1 per year)**				
Yes	39 (9.13)	39 (9.13)		78 (9.13)
No	388 (90.87)	388 (90.87)	1.00	776 (90.87)
**Self-rated health status**				
Poor / Very poor	39 (9.14)	55 (12.88)		94 (11.00)
Quite good	199 (46.60)	174 (40.75)		373 (43.68)
Good / Very good	189 (44.26)	198 (46.37)	0.1246	387 (45.32)
**PSA screening experience**				
Every year / Every 2 years	157 (36.77)	181 (42.39)		338 (39.58)
Every 4 years and less	62 (14.52)	66 (15.46)		128 (14.99)
Never	208 (48.71)	180 (42.15)	0.3469	388 (45.43)
**Digital rectal examination experience**				
Every year / Every 2 years	44 (10.30)	63 (14.76)		107 (12.53)
Every 4 years and less	113 (26.46)	108 (25.30)		221 (25.88)
Never	270 (63.23)	256 (59.95)	0.0015	526 (61.59)
**Colorectal cancer screening experience**				
Every 2 years	191 (44.73)	181 (42.39)		372 (43.56)
Less than every 2 years	87 (20.37)	81 (18.97)		168 (19.67)
Never	149 (34.89)	165 (38.64)	0.5225	314 (36.77)
**Know anyone with prostate cancer?**				
Yes	78 (18.27)	76 (17.80)		154 (18.03)
No	349 (81.73)	351 (82.20)	0.8587	700 (81.97)

Concerning screening behavior, 39.58% of the population declared performing PSA screening every year or every 2 years. Only 12.53% men underwent a digital rectal examination with the same frequency, and 61.59% had never received this clinical exam. Compared to prostate cancer screening, participation to organized colorectal cancer screening was higher (43.56%). Another screening attitude indicator was agreement with the question “*Do you ever examine your body to find whether there is something wrong?*” for which 9.49% checked “often” or “most of the time”. About 92% of respondents judged the questionnaire easy to very easy.

Table 2.

### Distribution of choices

The first screening alternative (i.e. screening test A) was chosen 1.480 times (frequency = 24.76% with video: 663 times without video: 817 times), the second screening alternative (i.e. screening test B) 1.604 times (frequency = 26.83% with video: 761 times without video: 843 times) and the opt-out option 2.894 times (frequency = 48.41% with video: 1.565 times without video: 1.329 times). Men using the video were more likely able to select the opt-out option “no screening option” (frequencies: 52.36% vs 44.46% *p* < 0.001).

Parameter estimations are detailed in Table 3. The coefficients under attribute represent importance of each attribute/screening characteristic in the final decision. Except for overdiagnosis and recommended frequency, all the attributes had the expected sign. Mortality by prostate cancer, false positive result, false negative result and out-of-pocket costs had a negative sign and were significant at the 5% level. Recommended frequency was not significant, so screening test frequency is not a major component prostate cancer screening decision. The overdiagnosis component had an unexpected positive sign and was statistically significant. In other words, men tend to attach more importance to an increase in overdiagnosis. The intercept was not significant, which means that the major components of screening choice are integrated as attributes. In other words, there is no missing component which influenced significantly screening decision.

**Table 3:** Men’s preferences for prostate cancer screening based on main effect logit model

***Attribute***	Estimates (N = 854*7)	
	***Coefficients***	***P-value***
***Constant***	0.1308	0.0999
***ASC***_***opt-ou***_	0.2671	< 0.0001
***PC mortality***	−76.9197	< 0.0001
***False positive***	−3.0764	< 0.0001
***False negative***	−21.3894	0.0002
***Overdiagnosis***	5.0361	0.0003
***Screening frequency***	0.00598	0.8165
***Out-of-pocket costs***	−0.0122	< 0.0001
***WTP***		
***Mortality reduction***	6.304.89	
***False positive***	252.16	
***False negative***	1753.23	
***Overdiagnosis***	−412.80	
***Statistical goodness of fit of model***		
***Pseudo R***^***2***^	0.06950	

Table 3.

### Willingness to pay

Willingness to pay (WTP) for several attributes is detailed in Table 3. Men were willing to pay for a reduction in prostate cancer mortality (6.304,89 € +/− 2.761,99) or false negative results (1.753,23 € +/− 1.054,65) more than for a reduction in false positive results (252,16 € +/− 68,54) and overdiagnosis (− 412,80 € +/− 233,00). Therefore, men are on average willing to pay 6.304,89 € to save a person’s life from prostate cancer. Because of large confidence intervals, WTP is more useful for hierarchizing preferences than for its monetary value.

### Effect of video

The effects of the video on the components are detailed in Table 4. As expected, viewing the video was associated with attributing value to the no-screening option. Men without access to the video were more likely to value the decrease in the risk of a false negative. Regarding the other risk attributes (i.e. mortality by prostate cancer, risk of false positive result, overdiagnosis), video access had no significant effect.

**Table 4:** Men’s preferences for prostate cancer screening based on main effect with interactions logit model

***Attribute***	Total (*N* = 779*7)	
	Coefficients	*P*-value
***Constant***	−0.1357	0.1087
***ASC***_***opt-ou***_	0.4164	< 0.0001
***PC mortality***	−76.7004	< 0.0001
***False positive***	−3.0794	< 0.0001
***False negative***	−20.0634	0.0011
***Overdiagnosis***	4.9651	0.0007
***Screening frequency***	0.00504	0.8536
***Out-of-pocket costs***	−0.0122	< 0.0001
***Effect of informative video***		
***Watching informative video***		
*no screening option	0.1737	< 0.0001
***No informative video access***		
*false negative	−12.1888	0.0046
***Interactions with individual characteristics***		
***Low level of health anxiety***		
*no screening option	0.1563	< 0.0001
*PC mortality	10.0525	0.0485
***High level of health anxiety***		
*no screening option	−0.2443	< 0.0001
*PC mortality	−13.2117	0.0080
***Irregular medical follow-up***		
*no screening option	0.1523	< 0.0001
***Absence of medical research***		
*no screening option	0.1882	< 0.0001
***Being involved in health decision process***		
*no screening option	0.1602	< 0.0001
***No experience of cancer screening (prostate or colorectal)***		
*no screening option	0.3930	< 0.0001
***Having experience of cancer screening (prostate or colorectal)***		
*PC mortality	−15.3020	0.0005
***Monthly income < 3000€***		
*out-of-pocket costs	0.00202	0.0222
***Worker status***		
*no screening option	−0.1747	< 0.0001
***Managerial status***		
*no screening option	−0.1140	< 0.0001
***Single/divorced/widower***		
*no screening option	0.0969	< 0.0001
Statistical goodness of fit of model		
***Pseudo R***^***2***^	0.1110	

### Investigation of heterogeneity

Several individual characteristics such as medical follow-up, information-seeking behaviour, integration in the health choice process, cancer screening experience, age, anxiety, health insurance, marital status, occupational category and highest level of education were selected in the choice models as interaction terms to investigate preference heterogeneity (Table 4). Most of the individual characteristics interacted with our dummy variable “no screening alternative”. A high level of health anxiety was associated with attributing value to screening alternatives and reduction of mortality due to prostate cancer. Irregular medical follow-up, involvement of men in the health decision process, passive information-seeking behaviour and no experience of cancer screening (i.e. at least one PSA assay or faecal blood test for prostate and colorectal cancer screening, respectively) had a negative effect on choosing a screening strategy. Moreover, men with experience of screening have a greater tendency to value a reduction in prostate cancer mortality. On the contrary, men living in a couple and with some occupational categories (i.e. workers, managerial and professional occupations) were more attracted by screening. Monthly income lower than the median of the sample reinforced the negative value attributed to out-of-pocket costs. The effect of the video persisted despite adjustment on individual characteristics.

Table 4.

## Discussion

The results of this DCE in men consulted about prostate cancer screening showed preferences in accordance with a priori expectations, excepted for overdiagnosis. Among the risk attributes, the number of prostate cancer deaths and the number of false negative results were the most important components of their screening decisions. In other stated preference studies, false negative risk was not included as an attribute, but reduction of mortality due to prostate cancer was more important than false positive and overdiagnosis/overtreatment risks [17, 18, 20], except in men aged from 40 to 49 years old in one study. This preference ranking could be due to a fear and anxiety relative to cancer. In most cases, men probably would not take the risk of a delayed prostate cancer diagnosis and risk missing the putative benefits of early treatment of any potential cancer. In a systematic review of qualitative studies on prostate cancer screening published in 2017, the authors found 13 studies which described prostate cancer screening as a survival imperative [35].

Another finding of this study is the unexpected sign of the overdiagnosis attribute. In stated preferences on prostate cancer screening, it was also considered as a positive argument in a French study [20] and negative in another one from the Netherlands [17]. Overdiagnosis could be considered positive in our analysis because of a misunderstanding of the term. Overdiagnosis is a relatively new and complex notion. Its definition is sometimes counterintuitive since cancer is perceived as a severe illness [36, 37]. Several studies investigated appropriation of this concept in the general population. In UK, about one third of 390 men or women aged 50 to 70 remembered having read or heard the term [38]. The rate was lower (7.7%) when participants were asked to give a definition of overdiagnosis. In prostate cancer, about 18% of US men with experience of a PSA assay declared being aware of the risk of overdiagnosis [39]. We assume that despite efforts to disseminate this notion through an informative video and stated preference instructions, some men in our sample may have misunderstood the term.

Another explanation of this unexpected sign is that some men may consider overdiagnosis as an opportunity to choose a less invasive treatment. They may wish to know as soon as possible if they have prostate cancer so that less invasive treatment is offered to them. This eventuality was identified during qualitative interviews in a previous part of this project about prostate cancer participation.

The video seemed to have a global effect on screening intention but no (or relatively little) effect on the value given to specific attributes. The reduction in stated participation is congruent with the findings of other studies assessing decision aids in prostate cancer screening [12]. Because the video covered wide-ranging topics, we assume that it is not sufficient in itself to grasp complex notions like risk components such as overdiagnosis. Information provided by the video should not replace that given by GPs. Rather, it could act as a starting point for fuller discussion with them.

### Strengths and weaknesses

This study is one of the first on prostate cancer screening participation to use a DCE methodology [40]. It is different from other DCE on men’s preferences, since it is the only one to consider every main benefit and risk of prostate cancer screening as attributes. It also has the largest population of respondents. Respondents were identified through a survey institute panel, were contacted by e-mail and were time-compensated. Although this inclusion strategy may have induced a selection bias, it was a way to be representative of our target population with the application of quotas. However, the representativeness of the participants sample could not be fully assessed because of a lack of non respondents data. Furthermore, we investigated men’s preferences with fictive choice scenarios. Some of these tasks presented are unrealistic. This could induce that some respondents did not consider unrealistic choice situations. However, in pre test phase of the questionnaire, nobody noticed difficulties with these unrealistic choice situations. In actual health situations, men’s behaviour might be different. For this reason, it is recommended to compare preferences stated in experimental settings with those observed in real-life conditions.

It is also one of the first study to test the effect of providing information on preferences [41]. Nevertheless, some of the parameters include may need to be modified in future studies using the DCE and the video.

The questionnaire was completed online and not face to face. It would have been useful to be able to assess the respondents’ attitude as they watched the video (e.g. lack of attention). We tried to maximize their attention during the video by obliging them to watch it in its entirety (i.e. fast-forward and next options were not available).

Finally, the time between the reception of information and the decision was not taken into account. The effect of the video could be modulated over time and together with a conversation with a health professional.

### Implications for clinicians and policymakers

Considering the preferences that the participants indicated, the act of viewing the video was not sufficient for all the ins and outs of screening to be understood. Yet the workload of GPs is increasing in France and their lack of time may be a reason why the benefits and risks of screening are not fully addressed. Therefore, the video could serve to facilitate the comprehension of complex terms and to trigger discussion with GPs.

## Conclusions

The participants attached importance to avoiding false negative results and prostate cancer mortality to the detriment of other risks of screening. More effort is needed to give men the opportunity to make informed choices because of the complexity of the benefit-risk ratio in prostate cancer screening.

## Data Availability

The datasets used and/or analysed during the current study are available from the corresponding author on reasonable request.

## Abbreviations

CAP: Cluster randomized trial of PSA testing for prostate cancer
DCE: Discrete choice experiment
ERSPC: European randomized study of screening for prostate cancer
GP: General practitioner
PLCO: Prostate, lung, colorectal and ovarian cancer screening trial
PSA: Prostate specific antigen
WTP: Willingness to pay
